# Refinement of immunizing antigens to produce functional blocking antibodies against the AniA nitrite reductase of *Neisseria gonorrhoeae*

**DOI:** 10.1371/journal.pone.0182555

**Published:** 2017-08-03

**Authors:** Lucy K. Shewell, Freda E.-C Jen, Michael P. Jennings

**Affiliations:** Institute for Glycomics, Griffith University, Gold Coast, Queensland, Australia; Instituto Butantan, BRAZIL

## Abstract

The emergence of multi-drug resistant *Neisseria gonorrhoeae* has generated an urgent need for novel therapies or a vaccine to prevent gonococcal disease. In this study we investigate the potential of targeting the surface exposed nitrite reductase, AniA, to block activity by producing functional blocking antibodies. AniA activity is essential for anaerobic growth and biofilm formation of *N*. *gonorrhoeae* and functional blocking antibodies may prevent colonisation and disease. Seven peptides covering regions adjacent to the active site were designed based on the AniA structure. Six of the seven peptide conjugates generated immune responses. Peptide 7, GALGQLKVEGAEN, was able to elicit antibodies capable of blocking AniA activity. Antiserum raised against the peptide 7 conjugate detected AniA in 20 *N*. *gonorrhoeae* clinical isolates. Recombinant AniA protein antigens were also assessed in this study and generated high-titre, functional blocking antibody responses. Peptide 7 conjugates or truncated recombinant AniA antigens have potential for inclusion in a vaccine against *N*. *gonorrhoeae*.

## Introduction

*Neisseria gonorrhoeae*, or the gonococcus, is a strictly human pathogen and the causative agent of the sexually transmitted disease gonorrhoea. In 2012, the World Health Organisation estimated that there were 106 million cases of gonorrhoea worldwide annually [1]. Currently there is no vaccine to prevent infection by *N*. *gonorrhoeae*. Moreover, there are now multiple reports of *N*. *gonorrhoeae* strains that are resistant to the last remaining first-line treatment option for gonorrhoea, the extended-spectrum cephalosporins [2–4]. The Centers for Disease Control and Prevention (CDC) have declared drug-resistant *N*. *gonorrhoeae* as an ‘immediate public-health threat that requires urgent and aggressive action’ [5]. There is, therefore, a pressing need for the development of new treatments or gonoccocal vaccines to prevent disease. There are a number of issues associated with the development of a vaccine for gonorrhoea including sequence variability in surface antigens, and the various mechanisms utilized by this pathogen to suppress and evade host immune responses [6].

In previous studies we have identified an outer membrane glycoprotein in the pathogenic *Neisseria*, the copper-containing nitrite reductase AniA [7, 8]. AniA is the major anaerobically induced outer membrane lipoprotein in *N*. *gonorrhoeae* [9, 10] and is essential for the growth and survival of *N*. *gonorrhoeae* under oxygen-limiting conditions [11]. It has also been demonstrated that an immune response is generated against AniA in response to gonococcal infection [12]. *N*. *gonorrhoeae* has been shown to form biofilms *in vitro* and *in vivo* during cervical infection which may be associated with persistent gonococcal infection in asymptomatic women [13]. Expression of AniA is highly upregulated during biofilm growth of the gonococcus [14, 15] and anaerobic respiration mediated by AniA is essential to normal biofilm formation and is widespread in the substratum of gonococcal biofilms. We showed that all strains of *N*. *gonorrhoeae* surveyed have a functional copy of the *aniA* gene, suggesting that AniA may be essential for the survival of the gonococcus [7]. AniA is post-translationally modified by glycosylation of two sites in the C-terminus and this modification is immunodominant. Removal of this glycan generates a non-native immune response directed against the AniA protein that produces antibodies capable of functional blocking [8]. Functional blocking antibodies may contribute to blocking effective colonisation and thereby transmission. Modelling suggests that even partial efficacy in preventing transmission may lead to an effective vaccine-based solution to gonococcal disease [16].

In this study we further investigate the potential of AniA as a vaccine candidate for *N*. *gonorrhoeae* by refining the antigens used for immunization with a peptide-based strategy and assess the resulting non-native immune response to generate antisera with functional blocking activity.

## Materials and methods

### Synthesis of peptides

The peptides were synthesized and conjugated to Keyhole Limpet Hemocyanin (KLH) by Mimotopes, Australia. The conjugated peptide sequences corresponding to the target AniA protein are: E_79_KTMKMDDGVEYRY_92_, N_118_NPSSTVPHNVDFH_131_, A_142_TFTAPGRTSTFSF_155_, A_168_VAPVGMHIANGM_180_, K_209_GKKGAQGLQPFD_221_, G_244_DNALKAKAGETV_256_, G_330_ALGQLKVEGAEN_342_.

### Ethical statement

All experimental work involving animals was approved by the Griffith University Animal Ethics Committee (Approval number: BDD/03/10) in accordance with the National Health and Medical Research Council (NHMRC) Australian Code for the Care and Use of Animals for Scientific Purposes and the Queensland Animal Care and Protection Act 2001. Rabbits were housed one per cage while mice were housed 5 per cage, with environmental enrichment including chew sticks, shredded newspaper and hay. Animals were monitored daily for signs of activity, coat appearance, breathing, movement, eating, drinking and alertness. Rabbits were sacrificed by anaesthetization for non-recovery using an AEC approved anaesthetic and mice were sacrificed by cervical dislocation and CO_2_ in a rising concentration with conformation of death.

### Immunization of rabbits

9 New Zealand white rabbits were immunized subcutaneously on day 0 with 100μg of peptides 1–7 conjugated to KLH or antigens 1 and 5 in phosphate buffered saline (PBS) combined 1:1 with Freund’s Complete Adjuvant (Sigma-Aldrich) in a final volume of 1ml. Rabbits were immunized again on days 21, 42, 63 and 84 with 100μg of peptides 1–7 conjugated to KLH or antigens 1 and 5 in PBS combined 1:1 with Freund’s Incomplete Adjuvant (Sigma-Aldrich) in a final volume of 1ml. Terminal bleeds were collected from each rabbit 21 days following the final immunization. The serum was harvested (post-immune serum) and stored at 4°C for immediate use or at -20°C. Pre-bleeds were collected on day -2 and the serum was collected (pre-immune serum) and stored at 4°C for immediate use or at -20°C.

### Immunization of mice

Groups of 10 female BALB/c mice were immunized subcutaneously with 10μg of peptides 1–7 conjugated to KLH or 5μg of antigens 1–8 in PBS combined 1:1 with Alhydrogel (Sigma-Aldrich) in a final volume of 200μl on day 0, 21 and 28. Terminal bleeds from each mouse were collected on day 42. The serum was harvested (post-immune serum) and stored at 4°C. Equal volumes of serum from all mice in each group were combined when pooled sera was used for assessment of immune response. Pre-bleeds were collected on day -2 and the serum was collected (pre-immune serum) and stored at 4°C.

### Bacterial strains and growth conditions

Bacterial strains used in this study are listed in Table 1. *N*. *gonorrhoeae* strains were grown on GC agar supplemented with 1% (v/v) IsoVitalex or in BHI broth supplemented with 10% (v/v) Levinthal’s base and 1% (v/v) IsoVitalex at 37°C with 5% CO_2_ for 16–18 hours. Oxygen-limited conditions for the growth of *N*. *gonorrhoeae* were created by growing cells in an appropriate tube completely filled with BHI broth supplemented with 10% (v/v) Levinthal’s base and 1% (v/v) IsoVitalex without shaking for 18–20 hours. Media was supplemented with 2mM sodium nitrite (NaNO_2_) to allow growth under oxygen-limited conditions. *E*. *coli* strains were grown in Luria-Bertani (LB) broth or on LB agar, supplemented with ampicillin at a concentration of 100μg ml^-1^ where appropriate, at 37°C.

**Table 1:** Plasmids, strains and primers used in this study.

Plasmid/strain/ primer	Description	Source/ Reference
**Plasmids**		
pGEM-T Easy	T-cloning vector, Amp^R^	Promega
pET-15b	Protein expression vector, N-terminal His-tag, Amp^R^	Novagen
aniA_pET-15b	*aniA* from *N*. *gonorrhoeae* 1291 cloned into pET-15b without a His-tag, Amp^R^	This study
**Strains**		
*E*. *coli* DH5α	Cloning strain	[41]
*E*. *coli* BL21 (DE3)	Protein expression strain	[42]
AniA-BL21	*E*. *coli* BL21 (DE3) transformed with aniA_pET-15b	This study
pET-BL21	*E*. *coli* BL21 (DE3) transformed with pET-15b	This study
*Neisseria gonorrhoeae* 1291	Originally isolated from a male patient with gonococcal urethritis	[43]
1291 *aniA*::*kan*	*aniA* is inactivated by insertion of a kanamycin resistance cassette	[7]
***N*. *gonorrhoeae* clinical isolates**		
02G0142	DGI*	[7]
90/G747	DGI*	[7]
98G1131	DGI*	[7]
88G285	DGI*	[7]
02D004	DGI*	[7]
02D156	DGI*	[7]
02W001	DGI*	[7]
02W006	DGI*	[7]
98D159	DGI*	[7]
97D040	DGI*	[7]
01D1052	MI^#^	[7]
01G1370	MI^#^	[7]
02G0427	MI^#^	[7]
02G1036	MI^#^	[7]
94G163	MI^#^	[7]
00G0794	MI^#^	[7]
01D064	MI^#^	[7]
01D100	MI^#^	[7]
97D059	MI^#^	[7]
96D551	MI^#^	[7]
**Primers**		
aniA_XbaI-F	TCTAGAAATAATTTTGTTTAACTTTAAGAAGGAGATATACCATGATTGCTTCC	This study
aniA_BamHI-R	GCGTCCGGATCCTTATTAATAAACGCTTTTTTC	This study

*DGI: Disseminated gonococcal infection

^#^: Asymptomatic carriage or mucosal gonorrhoeae infection (MI)

### Analysis of immunogenicity

#### ELISA

Wells of 96-well Maxisorp plates (NUNC) were coated with 100ng of purified recombinant AniA antigen 4 [8] in 100μl of coating buffer (0.5M carbonate/bicarbonate buffer, pH 9.6) overnight at 4°C or with 50μl of heat-inactivated (56°C for 1 hour) *N*. *gonorrhoeae* 1291 wild-type cells grown under oxygen-limited conditions or *N*. *gonorrhoeae* 1291 *aniA*::*kan* cells adjusted to an OD_600_ of 0.2 in PBS. Wells coated with whole cells were allowed to dry overnight in a laminar flow cabinet. Wells were blocked with 100μl of 5% BSA in PBS for 1 hour at room temperature before being washed 3 times with PBS-0.1% Tween-20 (PBS-T). Pre-immune or post-immune sera were two-fold serially diluted in PBS in triplicate to a final volume of 100μl and was incubated for 1.5 hours at room temperature. Wells were washed 4 times with PBS-T. 100μl of Polyclonal Goat Anti-Rabbit Immunoglobulin HRP-conjugated (DakoCytomation) or Polyclonal Goat Anti-Mouse Immunoglobulin HRP-conjugated (DakoCytomation) secondary antibody at a dilution of 1: 10 000 in PBS was added to each well and was incubated for 1 hour at room temperature. Wells were washed 4 times with PBS-T. TMB Single Solution substrate (Life Technologies) was added to each well and the reaction was stopped after 30 minutes by the addition of 0.25N HCl. The absorbance of each plate was measured at 450nm. Negative controls wells consisted of all ELISA reagents excluding pre-immune or post-immune sera. An absorbance value was considered positive if the absorbance at 450nm was greater than the mean absorbance of the negative control wells + 3 standard deviations. The antibody titer was determined as the highest serum dilution giving a positive absorbance value and is reported as the geometric mean titre. *aniA* mutant: wild-type titre ratios were determined by dividing the geometric mean titre against the wild-type by the geometric titre against *aniA*::*kan*.

#### SDS PAGE and western blotting

*N*. *gonorrhoeae* 1291 and clinical isolates grown under oxygen-limited conditions or *N*. *gonorrhoeae* 1291 *aniA*::*kan* cells grown under standard conditions were adjusted to an OD_600nm_ of ~2.5 in PBS for SDS-PAGE using NuPAGE 4–12% Bis-Tris polyacrylamide gels (Life Technologies). Pre-immune or post-immune sera were diluted appropriately in 5% skim milk powder in TBS-T as the primary antibody. Secondary antibodies used were anti-mouse IgG AP-conjugate or anti-rabbit IgG AP-conjugate (Sigma-Aldrich) at a 1: 10 000 in 5% skim milk powder in TBS-T. Antibody binding was detected with SigmaFAST NBT/BCIP tablets (Sigma-Aldrich) according to the manufacturer’s instructions.

### Construction of the *E*. *coli* BL21 (DE3) strain expressing recombinant AniA (AniA-BL21)

The DNA sequence encoding the entire *aniA* gene, including the signal sequence, was amplified using the primer set aniA_XbaI-F/aniA_BamHI-R (Table 1) with *N*. *gonorrhoeae* 1291 genomic DNA as template. The resulting PCR product was cloned into pGEM-T Easy and was verified by DNA sequencing before subcloning into the expression vector pET-15b to create the plasmid construct aniA_pET-15b. This construct allowed the expression of recombinant AniA without an N-terminal His-tag. aniA_pET-15b was transformed into electrocompetent *E*. *coli* BL21 (DE3) cells to create the strain AniA-BL21. Protein expression was confirmed with anti-AniA rabbit polyclonal antiserum [8].

### Whole cell ELISA

pET-BL21 and AniA-BL21 cells were grown in LB broth supplemented with ampicillin at a concentration of 100μg ml^-1^ at 37°C to an OD_600nm_ of ~0.2 before protein expression was induced with 0.1mM IPTG at 22°C for 3 hours. Cells were harvested, washed twice in PBS and adjusted to an OD_600_ of 0.2 in PBS. Wells were coated with 50μl of cell suspension and were allowed to dry overnight in a laminar flow cabinet. Wells were blocked with 100μl of 5% BSA in PBS for 1 hour at room temperature before being washed 3 times with PBS-0.1% Tween-20 (PBS-T). Antiserum raised against recombinant AniA antigen 4 [8] was two-fold serially diluted in PBS in triplicate starting at a dilution of 1: 1000 to a final volume of 100μl and was incubated for 1.5 hours at room temperature. Wells were washed 4 times with PBS-T. 100μl of Polyclonal Goat Anti-Rabbit Immunoglobulin HRP-conjugated (DakoCytomation) secondary antibody at a dilution of 1: 10 000 in PBS was added to each well and was incubated for 1 hour at room temperature. Wells were washed 4 times with PBS-T. ELISA titres were determined as described above.

### Trypsin digest of surface exposed proteins

pET-BL21 and AniA-BL21 cells were inoculated into LB broth supplemented with ampicillin at a concentration of 100μg ml^-1^ from a mid-log phase culture (OD_600nm_ = ~ 0.4) and grown at 37°C for 1 hour before protein expression was induced with 0.1mM IPTG at 22°C for 3 hours. Cells were harvested, washed twice in PBS and adjusted to give a final OD_600nm_ of 1. 100μl of cell suspension was combined with 0μg or 2μg of trypsin (Mass Spectrometry Grade, New England Biolabs) and incubated at 37°C for 60 mins. Samples of cell suspensions at t0 and at 60 mins (t60) were taken in triplicate for the determination of CFUs/ml. This was conducted to ensure that cell lysis was not occurring over the course of the assay, which would be indicated by a reduction in CFUs/ml. Differences between CFUs/ml at t0 and at t60 for each sample were assessed for statistical significance with Prism 5 software (GraphPad Software, Inc.) using a two-tailed unpaired Student’s *t-test*. *P* values <0.05 were considered significant. Trypsin digest reactions were terminated by the addition of SDS-PAGE sample buffer and incubation at 95°C for 5 mins. The susceptibility of recombinant AniA to proteolytic digestion with trypsin was assessed by western blot analysis with anti-AniA polyclonal rabbit serum as the primary antibody (1: 20 000) and anti-rabbit IgG AP-conjugate (Sigma-Aldrich) as the secondary antibody (1: 10 000).

### Nitrite reductase assays

#### Recombinant *E*. *coli* assays

AniA-BL21 and pET-BL21 cells were grown in LB broth supplemented with ampicillin at a concentration of 100μg ml^-1^ and 0.5mM CuSO_4_ at 37°C to an OD_600nm_ of ~0.2 before protein expression was induced with 0.1mM IPTG at 22°C overnight. Cells were harvested and washed twice in PBS containing 1% BSA and 27.8mM glucose and resuspended to give a final OD_600nm_ of 0.033. Cells were pre-incubated with pooled pre-immune or post-immune rabbit sera, heat-inactivated at 56°C for 30 minutes, at a dilution of 1: 20 at 37°C for 30 minutes with gentle agitation before the addition of the electron donor benzyl viologen to a final concentration of 100μM, the reducing agent sodium dithionite to a final concentration of 5mM and sodium nitrite to a final concentration of 400μM to begin the reaction in a final volume of 1.5ml. Reactions were incubated at 37°C. 150μl samples were removed at t0 and at 45 minutes (t45), immediately vortexed for 5 seconds to oxidise the benzyl viologen, placed in boiling water for 5 minutes to completely stop the reaction and were centrifuged at 4°C for 5 minutes. 100μl of the supernatant was transferred to 96-well plates and the amount of nitrite present was detected by the addition of Griess reagent (100μl of N-napthylenediamine and 50μl of 1% sulfanilamide). The absorbance was measured at 540nm and the concentration of nitrite present was calculated using standard curves. The concentration of nitrite was used to calculate the reaction rate in nmoles nitrite reduced min^-1^ ml^-1^ OD of cells^-1^. The average reaction rate obtained for pET-BL21 cells pre-incubated with a 1:20 dilution of pooled pre-immune serum or each post-immune serum was deducted from the reaction rate obtained for AniA-BL21 cells in the presence of that same serum to obtain the reported reaction rates. Results are shown as the mean of triplicate assays ±1SD from the mean. Data were analysed for statistical significance with Prism 5 software (GraphPad Software, Inc.) using a two-tailed unpaired Student’s *t*-test. *P* values <0.05 were considered significant.

#### *N*. *gonorrhoeae* 1291 assays

Nitrite reductase assays were performed as described in [8] with the following minor modifications: *N*. *gonorrhoeae* 1291 wild-type cells were grown under oxygen-limited conditions for 16–18 hours, cells were resuspended to give a final OD_600nm_ of 0.25, cells were incubated with pooled pre-immune or post-immune rabbit sera at a dilution of 1: 20, the final reaction volume was 1ml, 150μl samples were removed at t0 and at 45 minutes (t45), placed in boiling water for 5 minutes to completely stop the reaction and were centrifuged at 4°C for 5 minutes, the reaction rate is expressed as nmoles nitrite reduced min^-1^ ml^-1^ OD of cells^-1^. No nitrite reductase activity was observed for 1291 *aniA*::*kan* cells with either pre-immune or post-immune serum (data not shown). Results are shown as the mean of triplicate assays ±1SD from the mean. Data were analysed for statistical significance with Prism 5 software (GraphPad Software, Inc.) using a one-tailed unpaired Student’s *t*-test. *P* values <0.05 were considered significant.

## Results

### Design of synthetic peptides used for immunization

**Fig 1:**
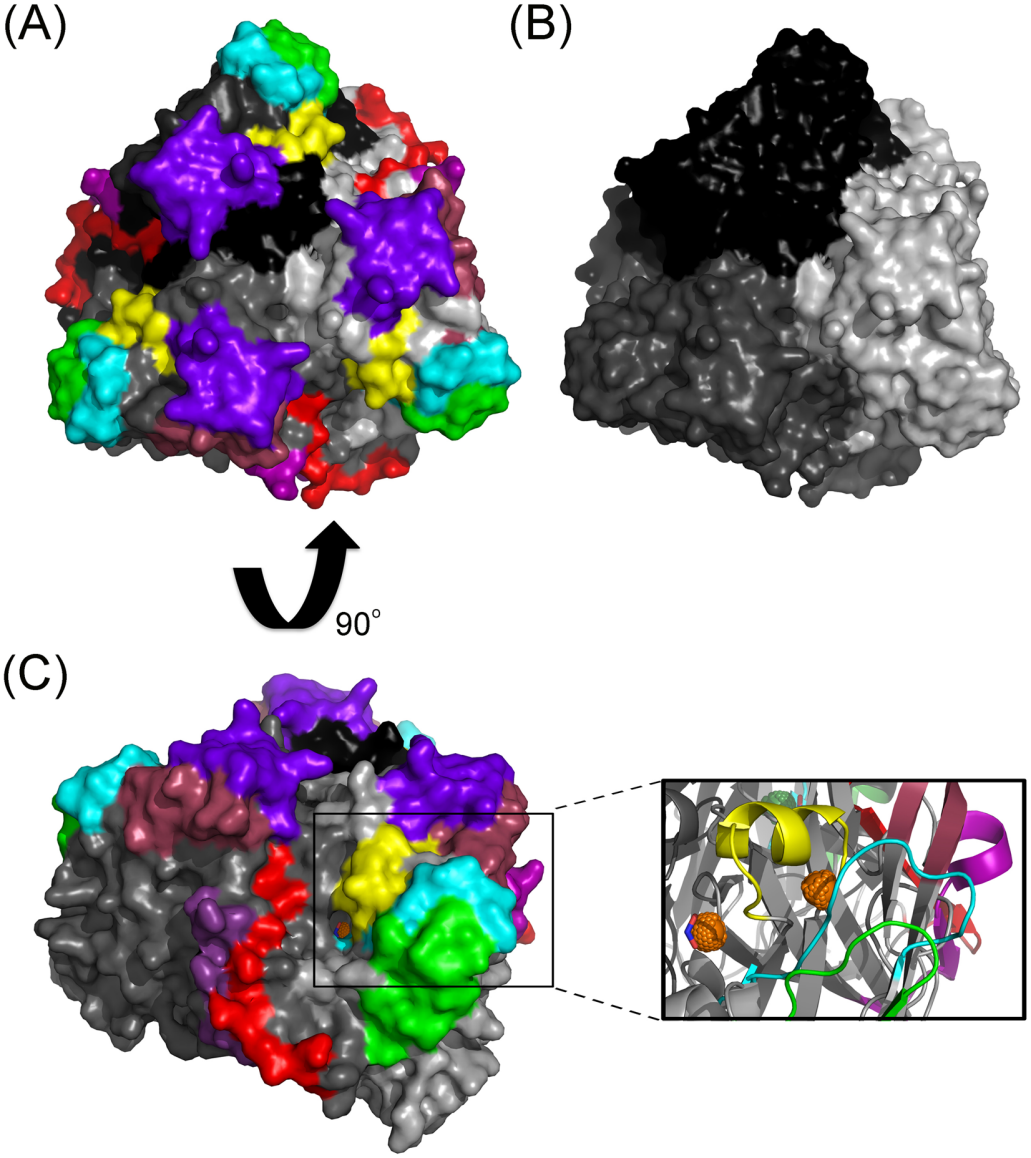
Crystal structure of the AniA trimer [17]. (A) The surface structure of the AniA trimer. The seven peptides that were designed to generate antibodies to inhibit AniA function are shown in seven different colours; 1 (maroon), 2 (cyan), 3 (green), 4 (yellow), 5 (purple), 6 (magenta), 7 (red). (B) Each monomer is shown in three different shades of grey. (C) The active sites of the AniA protein. Orange sphere indicates coppers (Cu). The inset shows the type I and type II Cu sites.

The AniA crystal structure shows that AniA forms a trimer [17] (as shown in Fig 1(A) and 1(B)). Each AniA monomer has a type I and type II copper site (Fig 1(C)) that are essential for electron transfer during nitrite reductase activity [17]. Electrons are first received at the type I copper site, then passed to a methionine residue located between the type I and type II site before passing on to the type II site. The type II copper site is where nitrite is converted to nitric oxide. Seven peptides adjacent to these key catalytic regions were designed to generate antibodies that may inhibit AniA activity. As shown in Fig 1(C), the peptides in cyan and green are close to the entrance of the type I cooper site. The yellow peptide is between the type I and type II copper sites. The purple, maroon and magenta peptides are the most surface exposed and antigenic regions of the AniA protein. Finally, the red peptide is next to the type II copper site of the adjacent AniA monomer. Antibodies binding to any of these regions have the potential to block AniA activity.

### Analysis of the immunogenicity of the synthetic peptides in rabbits and production of antisera

**Fig 2:**
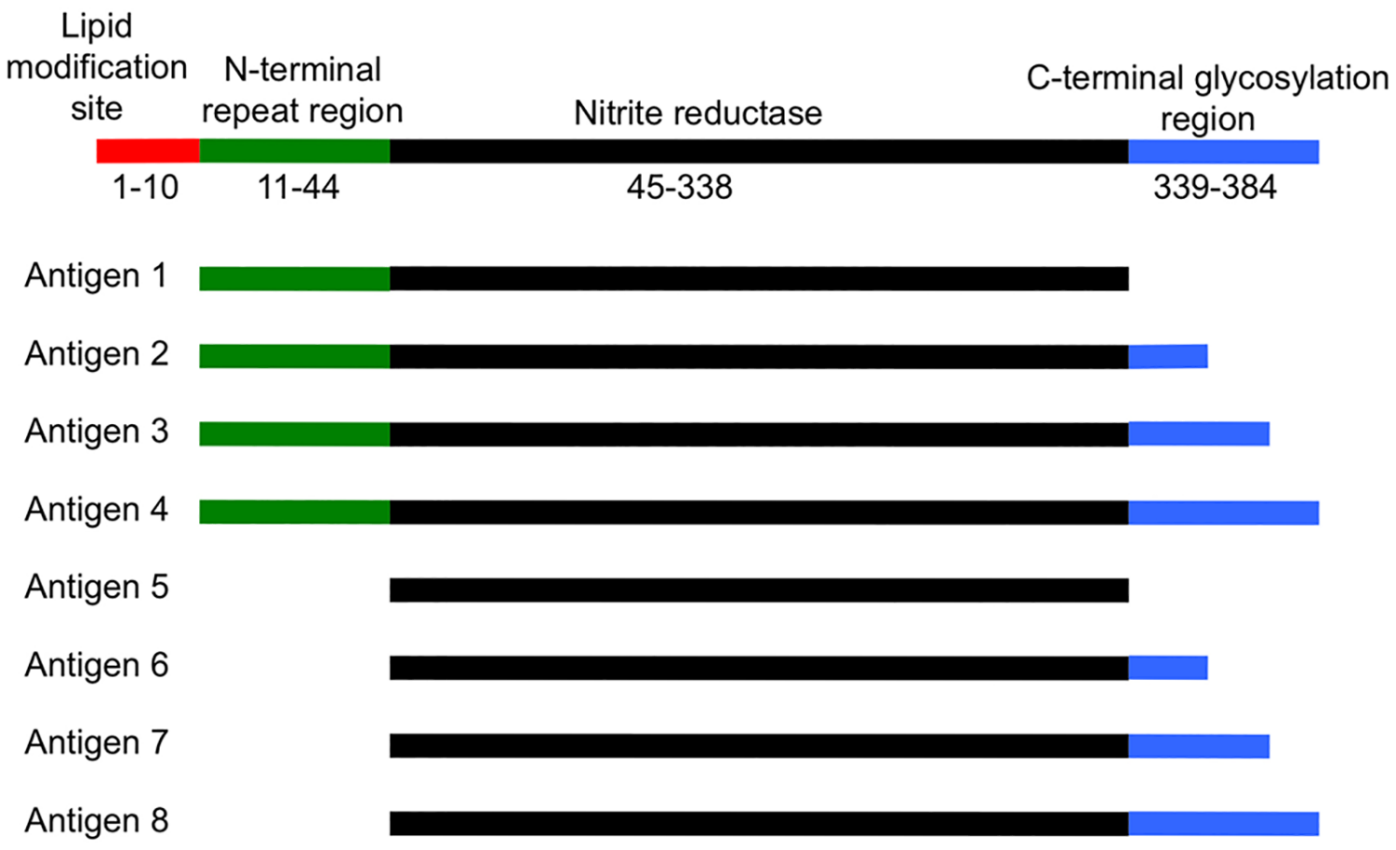
Diagrammatic representation of the full-length AniA protein from *N*. *gonorrhoeae* 1291 and of the recombinant AniA proteins (antigens 1–8) with various truncations of the N- and C-termini used for immunization. Lipid modification site = signal peptide containing a typical lipoprotein processing site (ALAAC), similar to sequences found in two other outer membrane gonococcal lipoproteins Lip/H.8 and Laz. This sequence is cleaved between Ala and Cys residues by signal peptidase II followed by N-terminal acylation of the Cys residue with palmitic acid (1–10, red); N-terminal repeat region = high in Ala and Pro content and contains several imperfect repeats of the AAEAP motif found in Lip and Laz [9] (11–44, green); Nitrite reductase = region included in the crystal structure of *N*. *gonorrhoeae* AniA and contains residues essential for copper binding at the type I site and type II site as well as the highly conserved active site residues Asp121 and His262 [17] (45–338, black); C-terminal glycosylation region, = contains the glycosylated serine residues, also contains four direct contiguous copies of a pentapeptide repeat, AASAP (339–384, blue).

**Fig 3:**
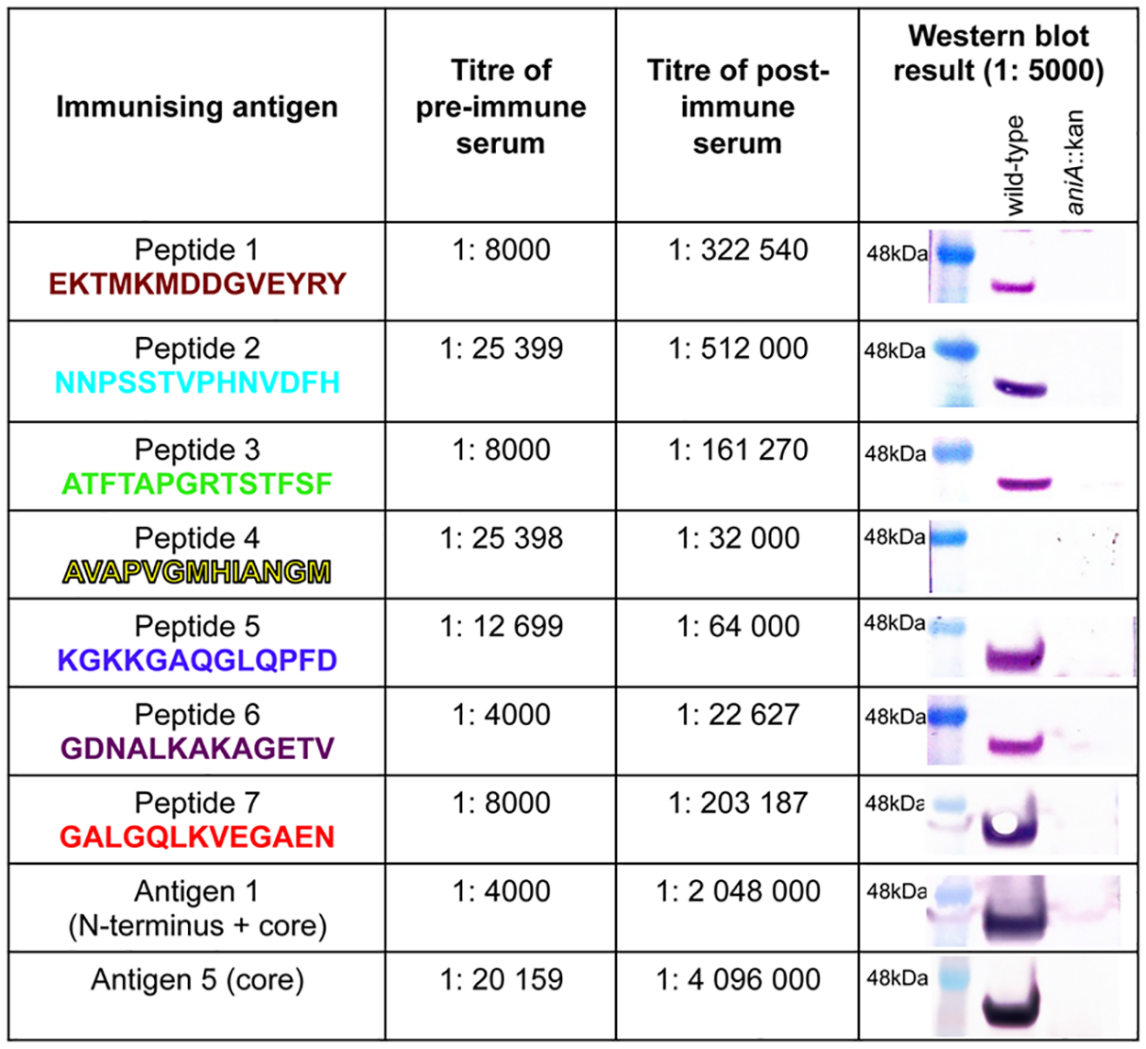
Analysis of the immunogenicity of the synthetic peptides and recombinant antigens 1 and 5 in rabbits. The titres of the pre-immune and post-immune sera from each rabbit were determined by ELISA against purified recombinant AniA (antigen 4 –see Fig 2 and [8]) and are shown as the geometric mean titre of triplicate assays. Western blot analyses were performed with post-immune sera from each rabbit against whole cell lysates of *N*. *gonorrhoeae* 1291 wild-type and *aniA*::kan. Pre-immune serum from each rabbit was also analysed by western blotting and no reactivity against AniA was observed (data not shown).

In order to produce antibodies against the seven peptides and assess the functional blocking activity of these antibodies, rabbits were immunized with peptides 1–7 conjugated to the carrier protein KLH. Rabbits were used for this purpose because the volume of antisera required for the assessment of functional blocking activity could not be obtained from mice. In addition, rabbits were immunized with recombinant AniA antigens 1 and 5, as previously described [8]. Antigen 1 consists of the 33 amino acid N-terminal repeat region of AniA, without the signal peptide which contains the lipid modification site, plus the AniA core region (amino acids 45–338) with the 45 amino acid C-terminal glycosylated region truncated. Antigen 5 consists of the AniA core region only (Fig 2). The immune response against each peptide was assessed by analysing the pre-immune serum and post-immune serum from each rabbit by western blotting and by ELISA against a recombinant, truncated form of AniA lacking the 10 amino acid N-terminal signal peptide and the glycosylation modifications (antigen 4 as described previously [8] and see Fig 2). All peptides except peptide 4 elicited an antibody response against AniA in the rabbits (Fig 3). The ELISA titres obtained were in general agreement with the reactivity observed via western blotting, except for antiserum against peptide 1. This may be because the antibodies generated against peptide 1 are better able to recognize conformational epitopes found in the native AniA structure compared to linear epitopes. Western blotting confirmed that the antibodies generated by immunization with the peptides were specific for AniA.

The antibody titres obtained by immunization with the peptides were considerably lower than the titres achieved by immunization with recombinant AniA antigens 1 and 5. This was reflected in the western blot results.

### Immunization with peptide 7 elicits functional blocking, cross-reactive antibodies

**Fig 4:**
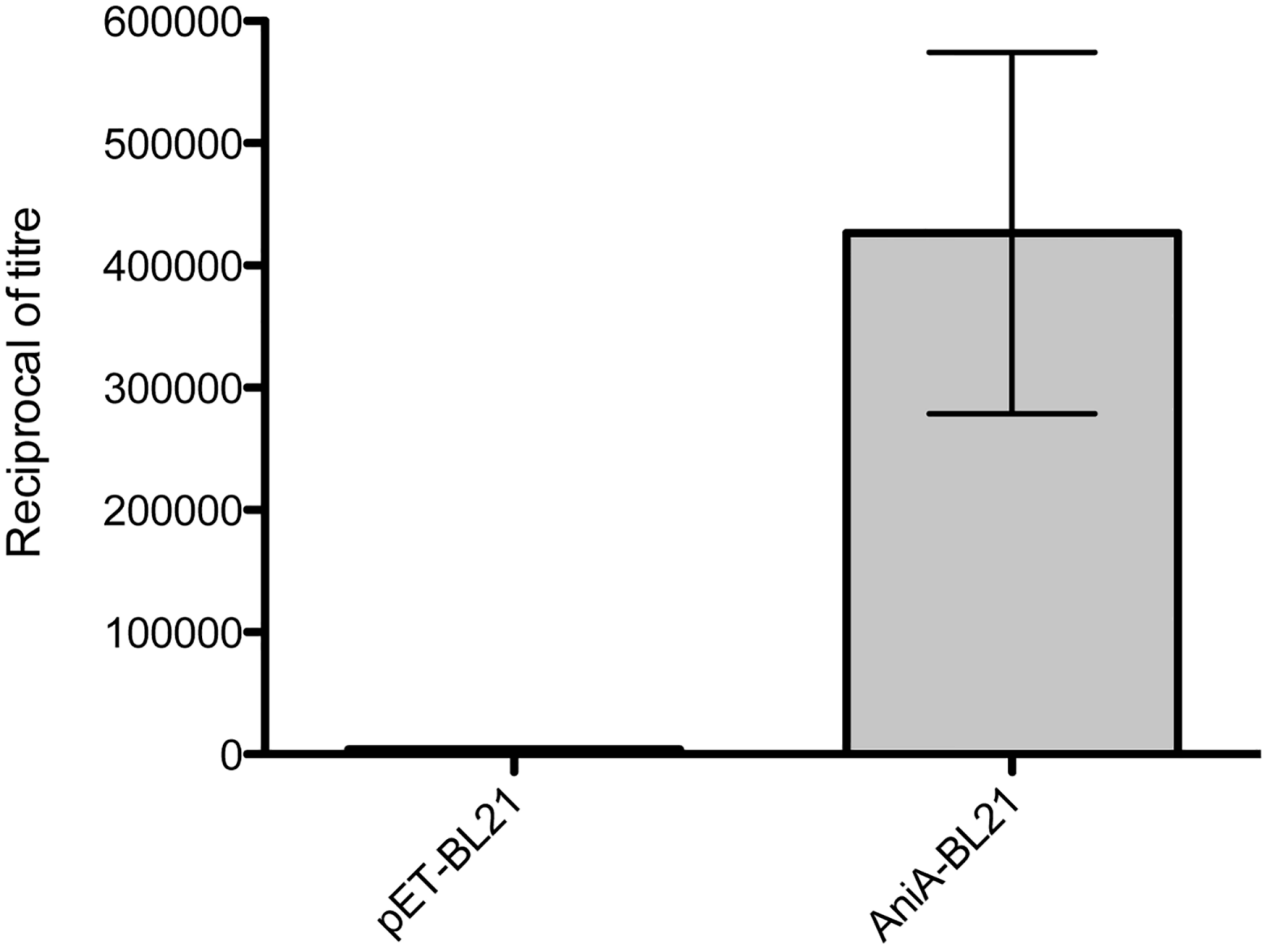
Analysis of the surface expression of recombinant AniA in AniA-BL21 by whole cell ELISA. Whole cell ELISA was performed on pET-BL21 and AniA-BL21 cells induced for protein expression at 22°C for 3 hours using antiserum raised against recombinant AniA antigen 4 [8] two-fold serially diluted starting at a dilution of 1: 1000. Polyclonal Goat Anti-Rabbit Immunoglobulin HRP-conjugated (DakoCytomation) was used as the secondary antibody at a dilution of 1: 10 000. Bars represent the mean from triplicate assays. Error bars represent ±1SD from the mean.

**Fig 5:**
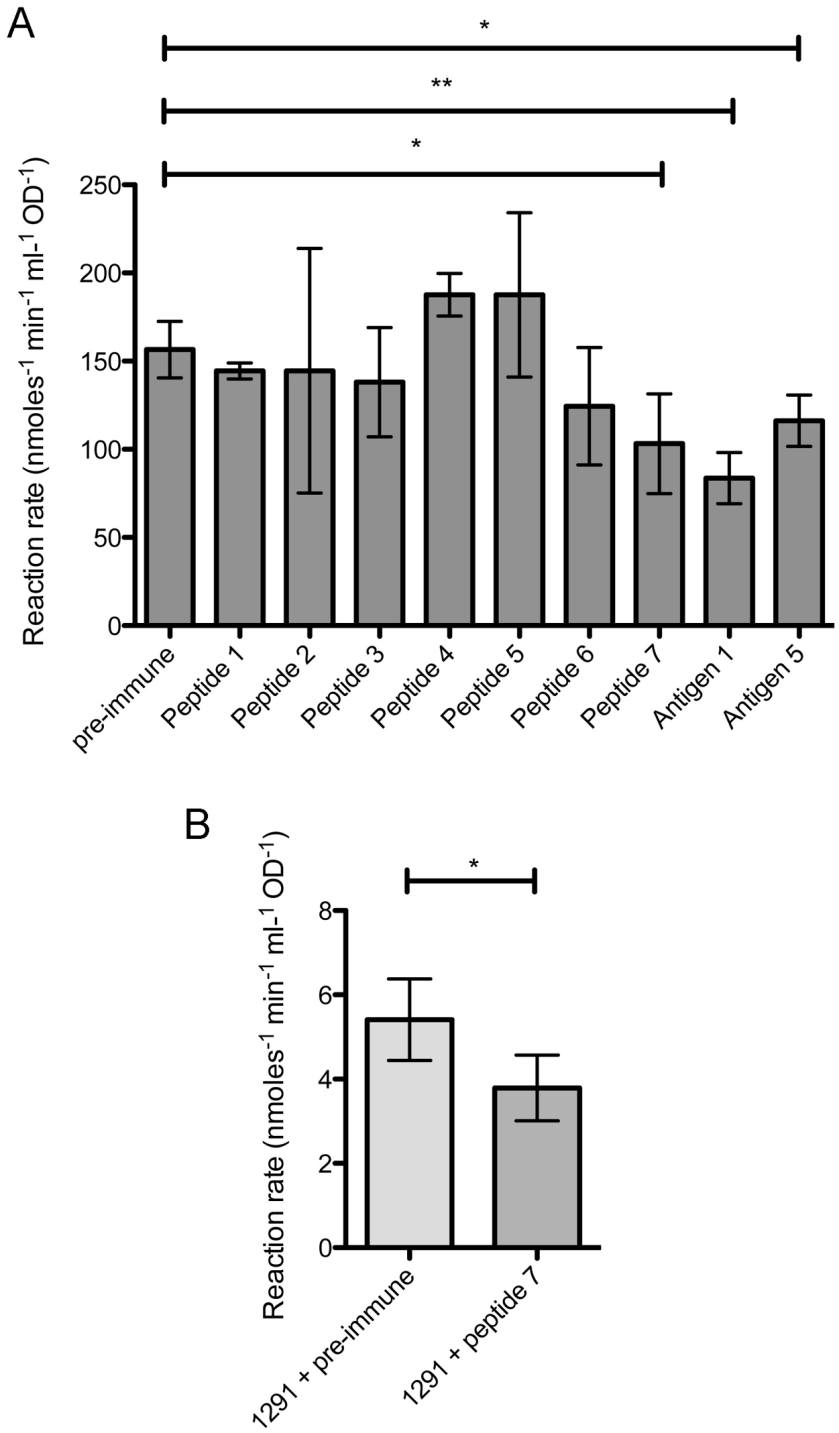
AniA activity blocking assays. (A) Bars represent AniA nitrite reductase activity expressed as the mean reaction rate (nmoles nitrite reduced min^-1^ ml^-1^ OD of cells^-1^) from triplicate assays of AniA-BL21 cells pre-incubated with a 1:20 dilution of pooled pre-immune sera or post-immune sera from each rabbit immunized with peptides 1–7, antigen 1 or antigen 5. Statistical significance was determined using a two-tailed unpaired Student’s *t-test*. * = *P*<0.05; ** = *P*<0.005. (B) Bars represent nitrite reductase activity from triplicate assays of *N*. *gonorrhoeae* whole cells pre-incubated with a 1:20 dilution of pooled pre-immune sera or post-immune serum from the rabbit immunized with peptide 7. Error bars represent ±1SD from the mean. Statistical significance was determined using a one-tailed unpaired Student’s *t-test*. * = *P*<0.05.

The post-immune serum from each rabbit immunized with peptides 1–7, as well as antigens 1 and 5, was assessed for the ability to inhibit AniA nitrite reductase activity. Inhibition of nitrite reductase activity was assessed using an *E*. *coli* BL21 (DE3) strain over-expressing full-length recombinant AniA from *N*. *gonorrhoeae* 1291 (AniA-BL21) without a His-tag. The expression of recombinant AniA on the cell surface in the AniA-BL21 strain was confirmed using whole cell ELISA (Fig 4) and by trypsin digest of surface exposed proteins (S1 Fig). The AniA-BL21 strain was used as this strain provided a more robust and sensitive assay compared to using whole *N*. *gonorrhoeae* cells grown under oxygen-limited conditions. To validate the method of assessment of functional blocking using the AniA-BL21 strain, antiserum raised against antigen 1 was included (Fig 5). In a previous study we showed that antiserum raised against antigen 1 was capable of significant inhibition of AniA nitrite reductase activity using whole *N*. *gonorrhoeae* 1291 cells [8]. Antiserum raised against antigen 5 was also assessed using this more robust and sensitive assay. Significant inhibition of nitrite reductase activity was observed with sera raised against antigen 1 and antigen 5, verifying our previous findings with this modified assay using the AniA-BL21 *E*. *coli* strain, and showing that antigen 5 can also elicit functional blocking antibodies.

Peptide 7 was the only peptide antigen that was able to elicit antibodies capable of blocking AniA activity. This antiserum was able to significantly inhibit the nitrite reductase activity of recombinant AniA expressed from the AniA-BL21 strain at a dilution of 1:20 (Fig 5(A)).

The antiserum raised against peptide 7 was further assessed for functional blocking antibodies against *N*. *gonorrhoeae* 1291 whole cells grown under oxygen-limited conditions. This antiserum significantly inhibited the nitrite reductase activity of *N*. *gonorrhoeae* 1291 cells at a dilution of 1:20 when compared to pre-immune serum (Fig 5(B)) further suggesting that peptide 7 can elicit functional blocking antibodies.

**Fig 6:**
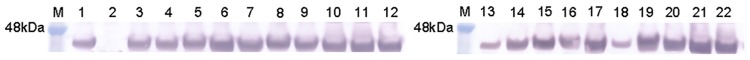
Antiserum raised against peptide 7 is cross-reactive with AniA from a range of *N*. *gonorrhoeae* clinical isolates. Antiserum from the rabbit immunized with peptide 7 was analysed by western blotting against cell lysates from a range of *N*. *gonorrhoeae* clinical isolates (Table 1) at a dilution of 1: 5000. M = molecular weight marker, 1: 1291, 2: 1291 *aniA*::kan, 3: 02G0142, 4: 90/G747, 5: 98G1131, 6: 88G285, 7: 02D004, 8: 02D156, 9: 02W001, 10: 02W006, 11: 98D159, 12: 97D040, 13: 01D1052, 14: 01G1370, 15: 02G0427, 16: 02G1036, 17: 94G163, 18: 00G0794, 19: 01D064, 20: 01D100, 21: 97D059, 22: 96D551.

The antibodies generated against peptide 7 were next assessed for cross-reactivity with AniA from a range of *N*. *gonorrhoeae* clinic isolates. These isolates had previously been shown to contain the *aniA* gene [7]. Using western blotting, the peptide 7 antiserum was found to react with AniA from all 20 strains of *N*. *gonorrhoeae* (Fig 6). These results suggest that the antibodies elicited by immunization with peptide 7 have functional blocking activity and are cross-reactive with a range of *N*. *gonorrhoeae* strains.

### Analysis of the murine humoral immune response to the recombinant AniA antigens and synthetic peptides

To determine whether the recombinant AniA antigens from our previous study could elicit a consistent immune response, groups of mice were immunized with these antigens using the immunization protocol described in [18]. Alhydrogel was used as the adjuvant as this is one of the few adjuvants licensed for use in humans [19]. The humoral immune response against AniA antigens 1–8 (see Fig 2 and [8]) in groups of 10 mice was assessed by whole cell ELISA against *N*. *gonorrhoeae* 1291 grown under oxygen-limited conditions and the 1291 *aniA* mutant (Table 2). Western blotting against cell lysates from *N*. *gonorrhoeae* 1291 wild-type and the *aniA* mutant verified the reactivity of these antibodies with AniA (S2 Fig). These results confirm that immunization with recombinant, non-glycosylated versions of AniA can elicit a reproducible, high-titre humoral immune response.

**Table 2:** Analysis of the murine immune response of the recombinant antigens 1–8. The titres of post-immune sera from individual mice in each group were determined by ELISA against *N*. *gonorrhoeae* 1291 wild-type and *aniA*:*kan* whole cells performed in triplicate. Titres are shown as the geometric mean titres of the means from the triplicate individual assays.

Immunising antigen	Titre against *aniA*::*kan*	Titre against wild-type	*aniA* mutant: wild-type titre ratio
Antigen 1	1: 11 143	1: 294 067	1:26.4
Antigen 2	1: 7879	1: 388 023	1:49.4
Antigen 3	1: 7518	1: 143 789	1:19.1
Antigen 4	1: 7518	1:67 080	1:8.9
Antigen 5	1: 6859	1: 207 937	1:30.3
Antigen 6	1: 10 631	1: 88 512	1:8.3
Antigen 7	1: 8830	1: 198 861	1:22.5
Antigen 8	1: 11 531	1: 249 731	1:21.7
Buffer + adjuvant	1: 6400	1: 5734	1:0.9

**Fig 7:**
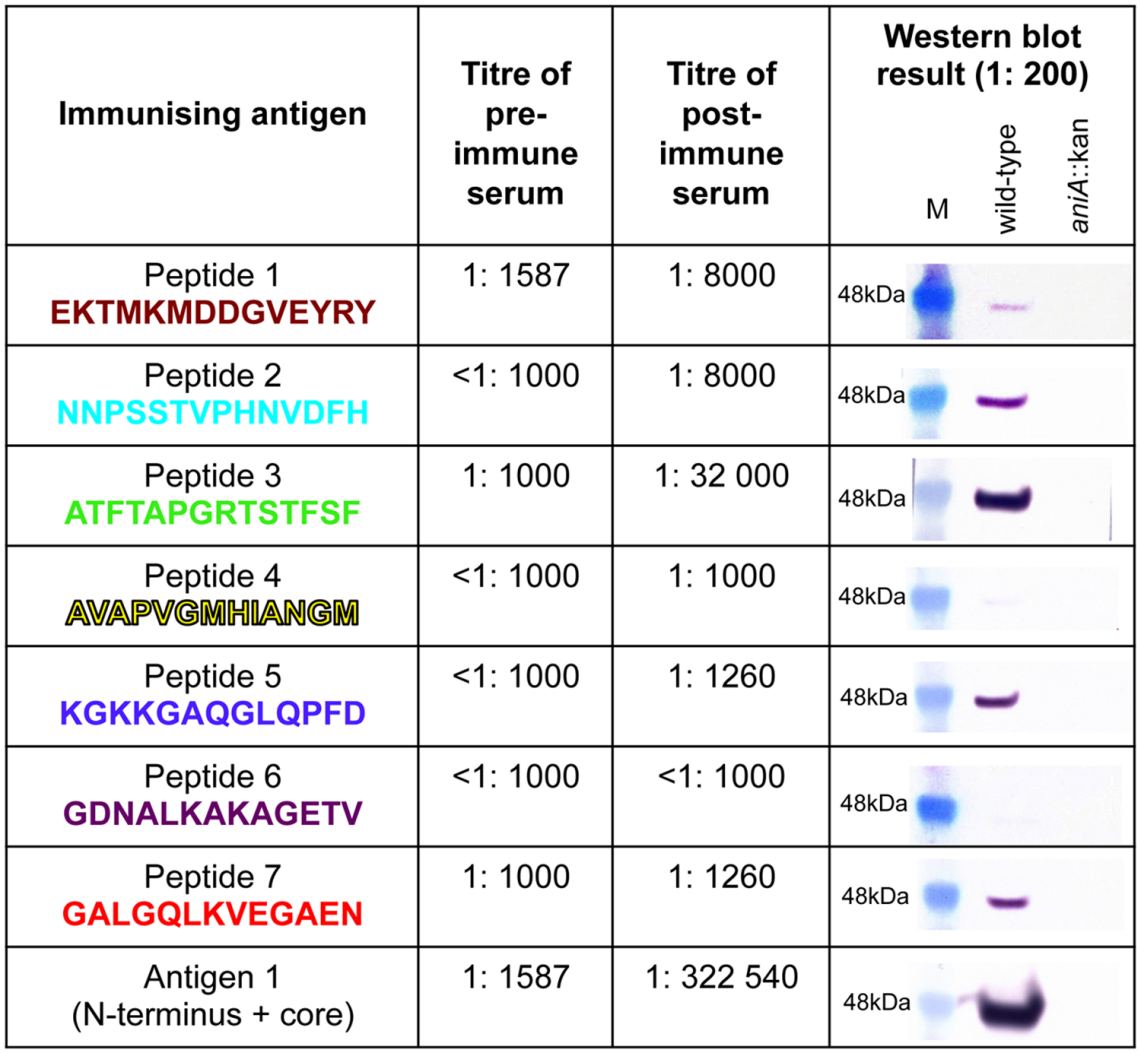
Analysis of the murine immune response against the synthetic peptides. The titres of pooled pre-immune and pooled post-immune sera from each group of mice were determined by ELISA against purified recombinant AniA (antigen 4 –see Fig 2 and [8]) and are shown as the geometric mean titre of triplicate assays. Western blot analyses were performed with pooled post-immune sera from each group of mice against whole cell lysates of *N*. *gonorrhoeae* 1291 wild-type and *aniA*::kan. Pooled pre-immune sera from each group of mice were also analysed by western blotting and no reactivity against AniA was observed (data not shown).

The immune response of the peptide conjugates was also assessed in groups of mice and compared to the antibody response generated against the recombinant AniA antigens, also in groups of mice. Groups of 10 mice were immunized with each of the 7 peptides conjugates, and again with antigen 1 for comparison. The humoral immune response against the synthetic peptides was assessed by ELISA against recombinant AniA antigen 4 (see Fig 2 and [8]) and by western blotting. Peptides 1, 2 and 3 elicited a humoral immune response against AniA that was detectable via both ELISA and western blotting (Fig 7). The western blot result for peptide 1 antisera was weaker than expected based on the ELISA titre obtained suggesting that antibodies generated against peptide 1 in mice are better able to recognize conformational epitopes. Peptides 5 and 7 generated very low titre antisera while the western blotting results showed fairly strong reactivity. This indicated that the antibodies elicited by these peptides recognize only linear epitopes. Peptides 4 and 6 did not elicit a detectable immune response against AniA in either the ELISA or via western blotting.

## Discussion

Low levels of systemic and local anti-gonococcal antibodies are detected in men and women who have been infected with *Neisseria gonorrhoeae*, but offer no protection from subsequent infections [20–23]. *N*. *gonorrhoeae* possesses multiple mechanisms to evade the host immune response. The ability of various surface structures, such as lipooligosaccharide (LOS) [24, 25], pili [26, 27] and Opa [28, 29], to undergo high frequency phase and antigenic variation during infection is one mechanism of immune avoidance. Furthermore, *N*. *gonorrhoeae* is able to suppress and withstand the host immune response using a multitude of sophisticated mechanisms. These proposed mechanisms include the down-regulation of the activation and proliferation of CD4 T cells via interactions with carcinoembryonic antigen-related cellular adhesion molecule (CEACAM)-1, inhibition of Th1/Th2-mediated adaptive immune responses, evasion of the host complement system and the resistance to killing by polymorphonuclear leukocytes [30–34]. Moreover, the female reproductive tract must accommodate a semi-allogenic fetus while at the same time confer protection against potential pathogens. Therefore, the typical methods for assessing the correlates of protection of a potential vaccine, such as serum bactericidal assays, may not be applicable to *N*. *gonorrhoeae*. These bacterial and host factors, in addition to the lack of an animal model that truly mimics human infection contribute to the difficulties in the development of a vaccine against *N*. *gonorrhoeae* [6].

In the past century three potential gonococcal vaccines have been tested in clinical trials. The first was a whole cell vaccine tested during the early 1900s [35]. In 1974 a partially autolyzed vaccine was tested [36], and in the 1990s a pilus-based vaccine was tested [37]. None of these vaccines were successful. These failed attempts combined with the lack of understanding of the correlates of protection indicate that novel strategies generating a non-native immune response are needed for the development of an efficacious vaccine against *N*. *gonorrhoeae*. We have previously shown that a non-native immune response can be generated by the immunization of rabbits with recombinant, non-glycosylated, truncated versions of the surface exposed, highly conserved nitrite reductase, AniA, of *N*. *gonorrhoeae* [8]. In this study we have shown that these recombinant antigens are also able to elicit a reproducible high-titre humoral immune response in multiple animals. By strategically designing synthetic peptides based on the AniA crystal structure, we were able to generate functional blocking antibodies from the immunization of rabbits with one particular peptide, peptide 7. We initially assessed the ability of the rabbit antisera to inhibit AniA nitrite reductase function with an assay using an *E*. *coli* strain over-expressing functional, recombinant AniA from *N*. *gonorrhoeae* 1291. Assays involving this *E*. *coli* strain were technically easier and more robust and sensitive than using *N*. *gonorrhoeae* 1291 whole cells for a number of reasons. The *E*. *coli* strain was far easier to grow and the levels of nitrite reductase activity were much higher in this over-expression strain compared to *N*. *gonorrhoeae*. Far more cells were required to obtain detectable nitrite reductase activity using 1291 cells grown under oxygen-limited conditions compared to the *E*. *coli* strain over-expressing recombinant AniA. Furthermore, we confirmed that recombinant AniA over-expressed in *E*. *coli* BL21 (DE3) was on the cell surface using both whole cell ELISA and trypsin digest of surface exposed proteins. This reflects the native situation in *N*. *gonorrhoeae*, where AniA is a lipoprotein anchored in the outer surface of the outer membrane. Therefore, this *E*. *coli* strain was used rather than purified enzyme in functional blocking assays as antibodies that are able to inhibit the activity of AniA presented on the cell surface are more likely to be effective at inhibiting AniA activity of the gonococcus during infection.

Previously, Edwards *et al*. showed that *N*. *gonorrhoeae* phospholipase D (PLD) enzyme activity could be inhibited by antisera raised against a peptide. PLD is a secreted effector that upregulates the pilus receptor CR3 on cervical epithelial cells and is key in promoting efficient colonization of these cells [38]. The peptide used for immunization corresponds to amino acids 181–195 of the active site of PLD and demonstrated the potential for a peptide to elicit functional blocking antibodies directed at a key enzymatic virulence factor [39]. Here we show that antibodies against peptide 7, which correspond to the residues at the surface entrance of the type II Cu site of AniA, where nitrite reductase activity takes place, showed inhibition of AniA activity. The mechanisms by which antibodies against peptide 7 could be inhibiting AniA function include blocking the nitrite entering into the type II Cu site, blocking electron donation from the exogenous electron donor (presumed to be a cytochrome) or antibody binding to AniA may change the overall conformation of AniA, thereby affecting enzyme function.

An ideal candidate vaccine antigen must be expressed in the majority of infecting strains and provide cross-reactive protection. The antiserum against peptide 7 was cross-reactive with a temporally and geographically diverse collection of 20 *N*. *gonorrhoeae* clinical isolates, verifying that AniA is expressed in a broad range of strains and that the protein portion is highly conserved making AniA a promising vaccine antigen. However, when the humoral immune response against peptide 7 was assessed in groups of mice, the titre obtained was relatively low when compared to the titres obtained from the immunization of groups of mice with the recombinant, truncated versions of AniA, antigens 1–8. Of the recombinant AniA antigens, immunization with recombinant protein antigen 1 and 5 reproducibly produces a high-titre immune response in antigenicity studies. These antigens can also generate antibodies that block nitrite reductase activity. Antigen 1 encompasses the N-terminus plus the core of AniA, while antigen 5 encompasses only the core. Both these truncated versions of AniA lack the C-terminal region, which contains the glycosylated residues, as well as four direct contiguous copies of a pentapeptide repeat, AASAP (see Fig 2). This repeat motif is also found in two other surface exposed gonococcal lipoproteins Lip (H.8) and Laz [9]. Naturally occurring human antibodies directed against the pentapeptide repeat motifs of the Lip and Laz lipoproteins are able to block killing of *N*. *meningitidis* by bactericidal antibodies [40]. This suggests that these pentapeptide repeat motifs may be immunogenic and may provide a subversive immune response. By removing not only the glycosylated residues, but also the C-terminal pentapeptide repeats from antigens 1 and 5, the antibody response may be further directed towards the core of the protein, thus explaining the ability of antibodies elicited by these antigens to block AniA function.

In conclusion, we have demonstrated the feasibility of a peptide-based antigen for functional blocking of AniA activity. Improvements in delivery and adjuvants for this peptide are required to improve the quality of the immune response. In comparison, recombinant AniA protein antigens 1 and 5 are superior to the synthetic peptide approach in that they generate high-titre, functional blocking antibodies suggesting that these recombinant proteins have potential for inclusion in a vaccine against *N*. *gonorrhoeae*.

## Supporting information

S1 Fig (A) Western blot analysis of whole, intact pET-BL21 and AniA-BL21 cells treated with trypsin using anti-AniA polyclonal rabbit serum. ** = Full-length AniA, * = Digested AniA. (B) CFUs/ml determined from samples taken at t0 and t60 for pET-BL21 and AniA-BL21. No significant differences were detected between the CFUs/ml at t0 and at 60mins (t60) for each of the samples as assessed by a two-tailed unpaired Student’s *t-test* indicating that no cell lysis had occurred over the course of the assay.
(TIF)

S2 Fig Analysis of the murine humoral immune response of the recombinant antigens 1–8 by western blot. Western blot analyses were performed with the pooled post-immune sera from each group of mice against whole cell lysates of *N*. *gonorrhoeae* 1291 wild-type and *aniA*::*kan*.
(TIF)

## References

[1] Organisation WH. Global incidence and prevalence of selected curable sexually transmitted infections– 2008. Geneva, Switzerland: 2012.

[2] Camara J, Serra J, Ayats J, Bastida T, Carnicer-Pont D, Andreu A, et al Molecular characterization of two high-level ceftriaxone-resistant Neisseria gonorrhoeae isolates detected in Catalonia, Spain. J Antimicrob Chemother. 2012;67(8):1858–60. doi: 10.1093/jac/dks162 . 22566592 10.1093/jac/dks162

[3] Ohnishi M, Golparian D, Shimuta K, Saika T, Hoshina S, Iwasaku K, et al Is Neisseria gonorrhoeae initiating a future era of untreatable gonorrhea?: detailed characterization of the first strain with high-level resistance to ceftriaxone. Antimicrob Agents Chemother. 2011;55(7):3538–45. doi: 10.1128/AAC.00325-11 ; 21576437 10.1128/AAC.00325-11 PMC3122416

[4] Scharbaai-Vazquez R, Gonzalez-Caraballo AL, Torres-Bauza LJ. Ceftriaxone-resistant Neisseria gonorrhoeae, Puerto Rico. Sex Transm Infect. 2014 doi: 10.1136/sextrans-2014-051716 . 25253760 10.1136/sextrans-2014-051716

[5] CDC. Antibiotic Resistance Threats in the United States, 2013. 2013 September 16, 2013. Report No.

[6] Edwards JL, Jennings MP, Apicella MA, Seib KL. Is gonococcal disease preventable? Importance of understanding immunity and pathogenesis in vaccine development. Critical Reviews of Microbiology (in press). 2015. 10.3109/1040841X.2015.1105782 PMC4958600 26805040

[7] Ku SC, Schulz BL, Power PM, Jennings MP. The pilin O-glycosylation pathway of pathogenic Neisseria is a general system that glycosylates AniA, an outer membrane nitrite reductase. Biochemical and biophysical research communications. 2009;378(1):84–9. doi: 10.1016/j.bbrc.2008.11.025 . 19013435 10.1016/j.bbrc.2008.11.025

[8] Shewell LK, Ku SC, Schulz BL, Jen FE, Mubaiwa TD, Ketterer MR, et al Recombinant truncated AniA of pathogenic Neisseria elicits a non-native immune response and functional blocking antibodies. Biochemical and biophysical research communications. 2013;431(2):215–20. doi: 10.1016/j.bbrc.2012.12.132 ; 23313483 10.1016/j.bbrc.2012.12.132 PMC4326246

[9] Hoehn GT, Clark VL. The major anaerobically induced outer membrane protein of Neisseria gonorrhoeae, Pan 1, is a lipoprotein. Infection and immunity. 1992;60(11):4704–8. ; 1398981 10.1128/iai.60.11.4704-4708.1992 PMC258221

[10] Clark VL, Campbell LA, Palermo DA, Evans TM, Klimpel KW. Induction and repression of outer membrane proteins by anaerobic growth of Neisseria gonorrhoeae. Infection and immunity. 1987;55(6):1359–64. ; 3106220 10.1128/iai.55.6.1359-1364.1987 PMC260520

[11] Mellies J, Jose J, Meyer TF. The Neisseria gonorrhoeae gene aniA encodes an inducible nitrite reductase. Mol Gen Genet. 1997;256(5):525–32. Epub 1997/12/31. . 9413436 10.1007/s004380050597

[12] Clark VL, Knapp JS, Thompson S, Klimpel KW. Presence of antibodies to the major anaerobically induced gonococcal outer membrane protein in sera from patients with gonococcal infections. Microb Pathog. 1988;5(5):381–90. Epub 1988/11/01. . 3148817 10.1016/0882-4010(88)90038-1

[13] Steichen CT, Shao JQ, Ketterer MR, Apicella MA. Gonococcal cervicitis: a role for biofilm in pathogenesis. J Infect Dis. 2008;198(12):1856–61. Epub 2008/11/01. doi: 10.1086/593336 ; 18973432 10.1086/593336 PMC2682323

[14] Falsetta ML, Bair TB, Ku SC, Vanden Hoven RN, Steichen CT, McEwan AG, et al Transcriptional profiling identifies the metabolic phenotype of gonococcal biofilms. Infect Immun. 2009;77(9):3522–32. Epub 2009/06/17. doi: 10.1128/IAI.00036-09 ; 19528210 10.1128/IAI.00036-09 PMC2737991

[15] Phillips NJ, Steichen CT, Schilling B, Post DM, Niles RK, Bair TB, et al Proteomic analysis of Neisseria gonorrhoeae biofilms shows shift to anaerobic respiration and changes in nutrient transport and outermembrane proteins. PLoS One. 2012;7(6):e38303 Epub 2012/06/16. doi: 10.1371/journal.pone.0038303 ; 22701624 10.1371/journal.pone.0038303 PMC3368942

[16] Craig AP, Gray RT, Edwards JL, Apicella MA, Jennings MP, Wilson DP, et al The potential impact of vaccination on the prevalence of gonorrhea. Vaccine. 2015;33(36):4520–5. doi: 10.1016/j.vaccine.2015.07.015 ; 26192351 10.1016/j.vaccine.2015.07.015 PMC4743649

[17] Boulanger MJ, Murphy ME. Crystal structure of the soluble domain of the major anaerobically induced outer membrane protein (AniA) from pathogenic Neisseria: a new class of copper-containing nitrite reductases. Journal of molecular biology. 2002;315(5):1111–27. doi: 10.1006/jmbi.2001.5251 . 11827480 10.1006/jmbi.2001.5251

[18] Peak IR, Srikhanta YN, Weynants VE, Feron C, Poolman JT, Jennings MP. Evaluation of truncated NhhA protein as a candidate meningococcal vaccine antigen. PloS one. 2013;8(9):e72003 doi: 10.1371/journal.pone.0072003 ; 24039731 10.1371/journal.pone.0072003 PMC3765393

[19] Brito LA, O'Hagan DT. Designing and building the next generation of improved vaccine adjuvants. Journal of controlled release: official journal of the Controlled Release Society. 2014;190:563–79. doi: 10.1016/j.jconrel.2014.06.027 . 24998942 10.1016/j.jconrel.2014.06.027

[20] Fox KK, Thomas JC, Weiner DH, Davis RH, Sparling PF, Cohen MS. Longitudinal evaluation of serovar-specific immunity to Neisseria gonorrhoeae. American journal of epidemiology. 1999;149(4):353–8. . 10025478 10.1093/oxfordjournals.aje.a009820

[21] Hedges SR, Mayo MS, Mestecky J, Hook EW 3rd, Russell MW. Limited local and systemic antibody responses to Neisseria gonorrhoeae during uncomplicated genital infections. Infection and immunity. 1999;67(8):3937–46. ; 10417159 10.1128/iai.67.8.3937-3946.1999 PMC96675

[22] Hedges SR, Sibley DA, Mayo MS, Hook EW 3rd, Russell MW. Cytokine and antibody responses in women infected with Neisseria gonorrhoeae: effects of concomitant infections. The Journal of infectious diseases. 1998;178(3):742–51. . 9728543 10.1086/515372

[23] Ross JD, Moyes A, Young H. Serovar specific immunity to Neisseria gonorrhoeae: does it exist? Genitourinary medicine. 1995;71(6):367–9. ; 8566975 10.1136/sti.71.6.367 PMC1196106

[24] Danaher RJ, Levin JC, Arking D, Burch CL, Sandlin R, Stein DC. Genetic basis of Neisseria gonorrhoeae lipooligosaccharide antigenic variation. Journal of bacteriology. 1995;177(24):7275–9. ; 8522539 10.1128/jb.177.24.7275-7279.1995 PMC177611

[25] Apicella MA, Shero M, Jarvis GA, Griffiss JM, Mandrell RE, Schneider H. Phenotypic variation in epitope expression of the Neisseria gonorrhoeae lipooligosaccharide. Infection and immunity. 1987;55(8):1755–61. ; 2440807 10.1128/iai.55.8.1755-1761.1987 PMC260597

[26] Seifert HS, Wright CJ, Jerse AE, Cohen MS, Cannon JG. Multiple gonococcal pilin antigenic variants are produced during experimental human infections. The Journal of clinical investigation. 1994;93(6):2744–9. doi: 10.1172/JCI117290 ; 7911129 10.1172/JCI117290 PMC294533

[27] Swanson J, Robbins K, Barrera O, Corwin D, Boslego J, Ciak J, et al Gonococcal pilin variants in experimental gonorrhea. The Journal of experimental medicine. 1987;165(5):1344–57. ; 3106555 10.1084/jem.165.5.1344 PMC2188307

[28] Jerse AE, Cohen MS, Drown PM, Whicker LG, Isbey SF, Seifert HS, et al Multiple gonococcal opacity proteins are expressed during experimental urethral infection in the male. The Journal of experimental medicine. 1994;179(3):911–20. ; 8113683 10.1084/jem.179.3.911 PMC2191399

[29] Swanson J, Barrera O, Sola J, Boslego J. Expression of outer membrane protein II by gonococci in experimental gonorrhea. The Journal of experimental medicine. 1988;168(6):2121–9. ; 3143800 10.1084/jem.168.6.2121 PMC2189168

[30] Liu Y, Liu W, Russell MW. Suppression of host adaptive immune responses by Neisseria gonorrhoeae: role of interleukin 10 and type 1 regulatory T cells. Mucosal immunology. 2014;7(1):165–76. doi: 10.1038/mi.2013.36 ; 23757303 10.1038/mi.2013.36 PMC3812424

[31] Liu Y, Russell MW. Diversion of the immune response to Neisseria gonorrhoeae from Th17 to Th1/Th2 by treatment with anti-transforming growth factor beta antibody generates immunological memory and protective immunity. mBio. 2011;2(3):e00095–11. doi: 10.1128/mBio.00095-11 ; 21610119 10.1128/mBio.00095-11 PMC3101786

[32] Boulton IC, Gray-Owen SD. Neisserial binding to CEACAM1 arrests the activation and proliferation of CD4+ T lymphocytes. Nature immunology. 2002;3(3):229–36. doi: 10.1038/ni769 . 11850628 10.1038/ni769

[33] Zughaier SM, Kandler JL, Shafer WM. Neisseria gonorrhoeae modulates iron-limiting innate immune defenses in macrophages. PloS one. 2014;9(1):e87688 doi: 10.1371/journal.pone.0087688 ; 24489950 10.1371/journal.pone.0087688 PMC3905030

[34] Johnson MB, Criss AK. Resistance of Neisseria gonorrhoeae to neutrophils. Frontiers in microbiology. 2011;2:77 doi: 10.3389/fmicb.2011.00077 ; 21747795 10.3389/fmicb.2011.00077 PMC3128980

[35] Eyre JWH, Stewart BH. The treatment of gonococcus infections by vaccines. Lancet. 1909;174:76–81.

[36] Greenberg L, Diena BB, Ashton FA, Wallace R, Kenny CP, Znamirowski R, et al Gonococcal vaccine studies in Inuvik. Canadian journal of public health = Revue canadienne de sante publique. 1974;65(1):29–33. . 4205640

[37] Boslego JW, Tramont EC, Chung RC, McChesney DG, Ciak J, Sadoff JC, et al Efficacy trial of a parenteral gonococcal pilus vaccine in men. Vaccine. 1991;9(3):154–62. . 1675029 10.1016/0264-410x(91)90147-x

[38] Edwards JL, Entz DD, Apicella MA. Gonococcal phospholipase d modulates the expression and function of complement receptor 3 in primary cervical epithelial cells. Infection and immunity. 2003;71(11):6381–91. doi: 10.1128/IAI.71.11.6381-6391.2003 ; 14573659 10.1128/IAI.71.11.6381-6391.2003 PMC219594

[39] Edwards JL, Apicella MA. Neisseria gonorrhoeae PLD directly interacts with Akt kinase upon infection of primary, human, cervical epithelial cells. Cellular microbiology. 2006;8(8):1253–71. doi: 10.1111/j.1462-5822.2006.00707.x . 16882030 10.1111/j.1462-5822.2006.00707.x

[40] Ray TD, Lewis LA, Gulati S, Rice PA, Ram S. Novel blocking human IgG directed against the pentapeptide repeat motifs of Neisseria meningitidis Lip/H.8 and Laz lipoproteins. Journal of immunology. 2011;186(8):4881–94. doi: 10.4049/jimmunol.1003623 ; 21402895 10.4049/jimmunol.1003623 PMC3125605

[41] Hanahan D. Studies on transformation of Escherichia coli with plasmids. Journal of molecular biology. 1983;166(4):557–80. . 6345791 10.1016/s0022-2836(83)80284-8

[42] Studier FW, Moffatt BA. Use of bacteriophage T7 RNA polymerase to direct selective high-level expression of cloned genes. Journal of molecular biology. 1986;189(1):113–30. . 3537305 10.1016/0022-2836(86)90385-2

[43] Apicella MA. Antigenically distinct populations of Neisseria gonorrhoeae: isolation and characterization of the responsible determinants. The Journal of infectious diseases. 1974;130(6):619–25. . 4139222 10.1093/infdis/130.6.619

