# Supplementary figures and images for: Refinement of immunizing antigens to produce functional blocking antibodies against the AniA nitrite reductase of Neisseria gonorrhoeae
Source: PLoS One. 2017 Aug 3;12(8):e0182555. doi: 10.1371/journal.pone.0182555 (PMC5542605; doi:10.1371/journal.pone.0182555)

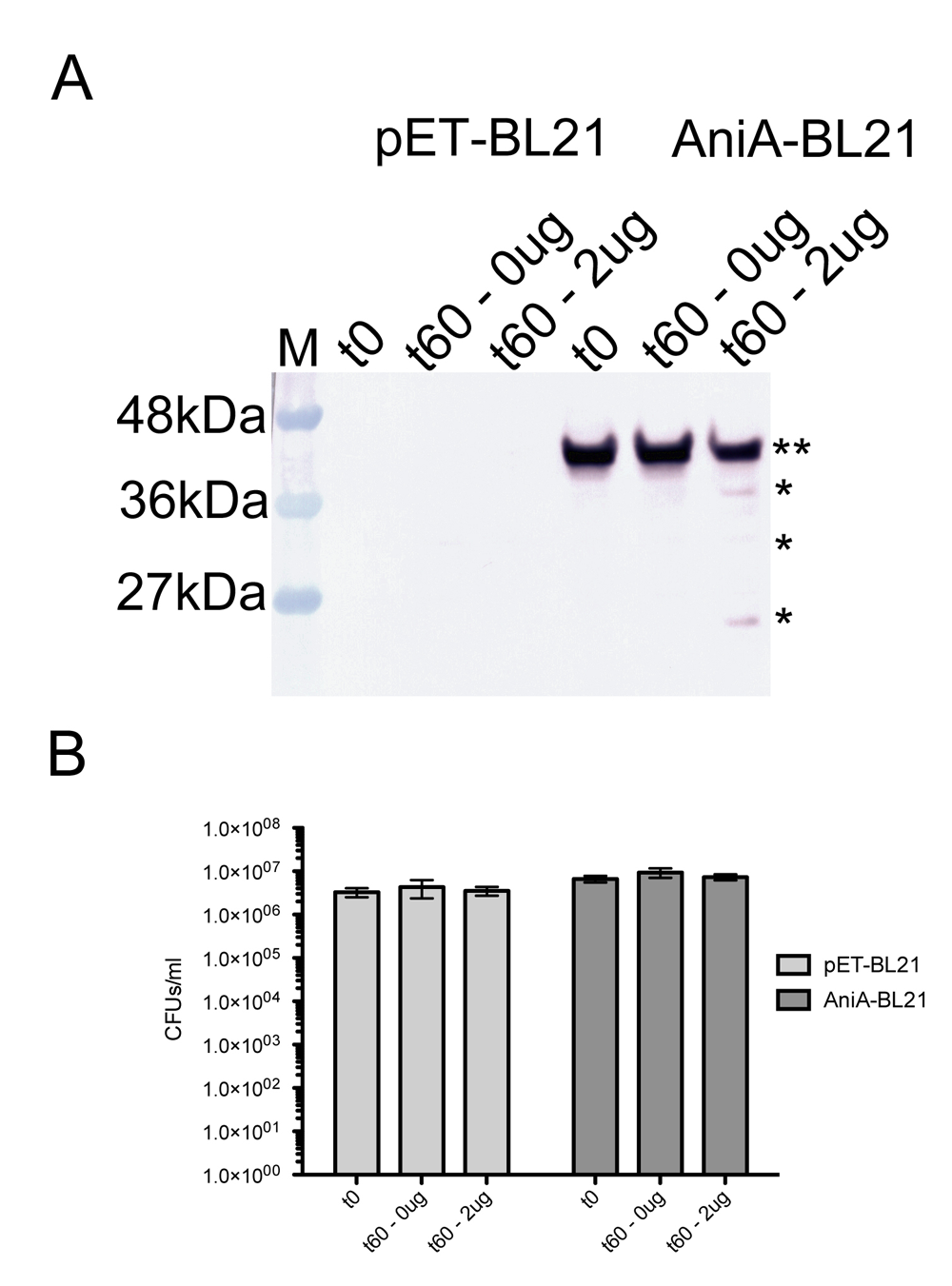

Supplement: S1 Fig — (A) Western blot analysis of whole, intact pET-BL21 and AniA-BL21 cells treated with trypsin using anti-AniA polyclonal rabbit serum. ** = Full-length AniA, * = Digested AniA. (B) CFUs/ml determined from samples taken at t0 and t60 for pET-BL21 and AniA-BL21. No significant differences were detected between the CFUs/ml at t0 and at 60mins (t60) for each of the samples as assessed by a two-tailed unpaired Student’s t-test indicating that no cell lysis had occurred over the course of the assay. (TIF) [file pone.0182555.s001.tif]

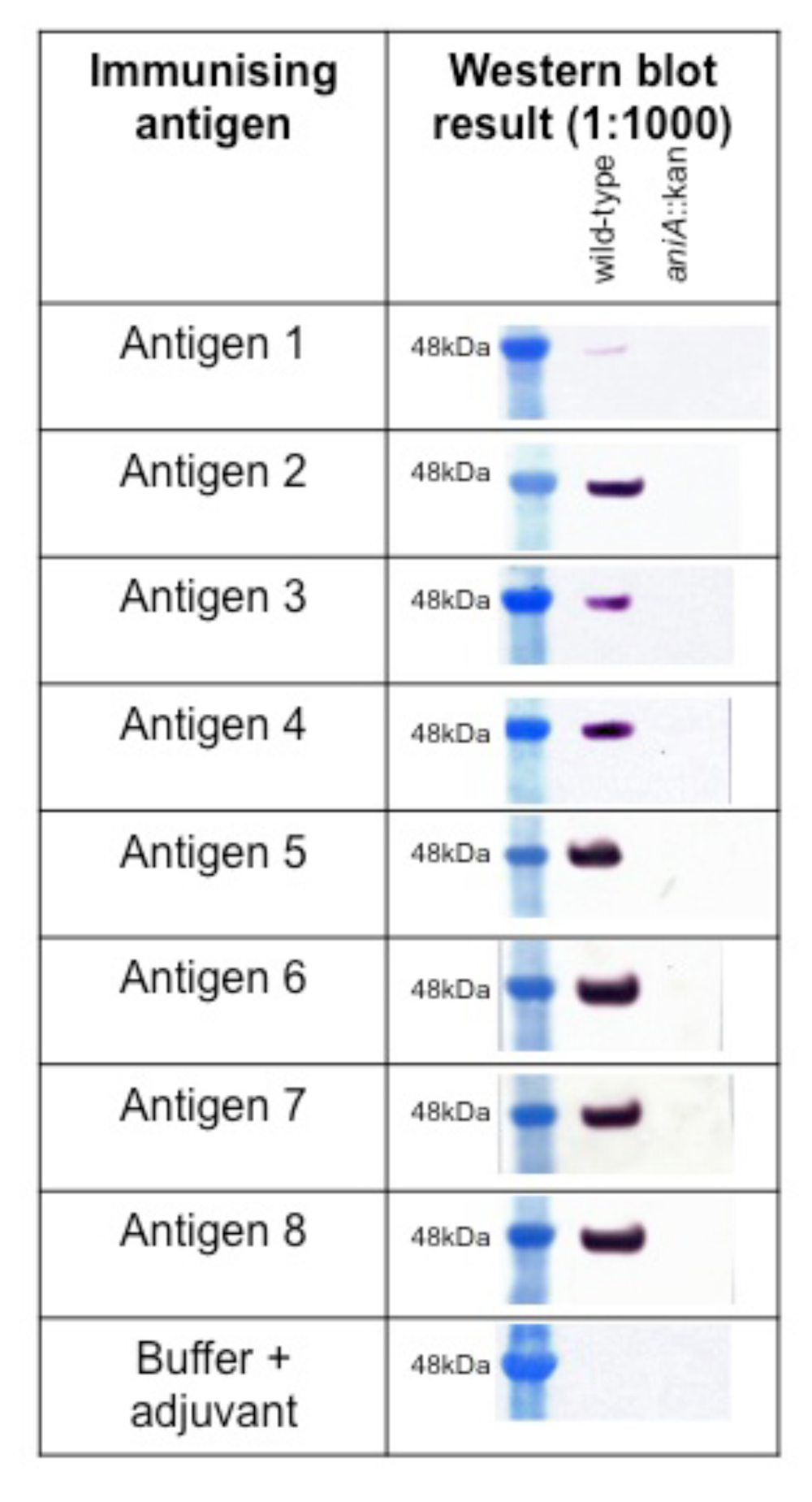

Supplement: S2 Fig — Western blot analyses were performed with the pooled post-immune sera from each group of mice against whole cell lysates of N. gonorrhoeae 1291 wild-type and aniA::kan. (TIF) [file pone.0182555.s002.tif]
